# COVID-19 vaccine hesitancy among health providers at Kenyatta National Teaching and Referral Hospital Nairobi-Kenya

**DOI:** 10.11604/pamj.2024.49.31.44719

**Published:** 2024-10-10

**Authors:** Phelix Okello, Vallery Ogello, Nicholas Thuo, Stephen Gakuo, Paul Mwangi, Peter Mogere, Paul Mutua, Harrison Mwenda, Linnet Ongeri, John Kinuthia, Nelly Mugo, Kenneth Ngure

**Affiliations:** 1 Partners in Health Research and Development, Center for Clinical Research, Kenya Medical Research Institute, Nairobi, Kenya,; 2 Kenyatta National Hospital, Nairobi, Kenya,; 3 Kenya Medical Research Institute, Nairobi, Kenya,; 4 School of Public Health Department, Jomo Kenyatta University of Agriculture and Technology, Nairobi, Kenya

**Keywords:** COVID-19 Vaccine, hesitancy, healthcare providers, Kenya

## To the editors of the Pan African Medical Journal

Health providers' delay of acceptance or refusal to receive the vaccine may slow down COVID-19 mass vaccination. Therefore, understanding health providers' COVID-19 vaccine concerns can provide critical insights to optimize the success of the vaccine rollout program as they are a trusted and important source of health information to patients and the general population [1]. We conducted a phenomenological study to understand the reasons for health providers' COVID-19 vaccine hesitancy at Kenyatta National Teaching and Referral Hospital, Kenya.

Between April to August 2021, we conducted in-depth interviews (IDIs) among providers at Kenyatta National Referral Hospital, Kenya. We employed stratified purposive sampling to enroll clinical care providers (medical officers, clinical officers, nurses), and pharmacy staff (pharmacists and pharmaceutical technologists). Eligible participants were those actively providing health care services at either the infectious disease unit (n=20) or non-infectious disease unit (n=40) at the Kenyatta National Hospital, willing and able to provide informed consent.

All interviews were conducted in English by experienced social scientists and transcribed verbatim. The social scientists developed a semi-structured interview guide to explore health providers' and colleagues' reasons for COVID-19 vaccine hesitancy, and suggestions to improve vaccine uptake among them and the general population. Experienced social scientists (PO, and VO), reviewed the interview transcripts to ensure consistency, clarity, and completeness, and subsequently coded supported by Dedoose software (version 8.3.35) (Sociocultural Research Consultants, LLC, Los Angeles, CA, USA), a web-based application for managing, analyzing, and presenting our qualitative data [2]. The two social scientists developed a thematic coding framework based on the topics covered in the interview guide. We used deductive and inductive content analytic approaches to identify emerging themes, and organize our findings within the 5c model of factors influencing vaccine hesitancy and acceptance including; confidence, convenience/constraints, complacency, calculation, and collective response [3], as in Table 1.

**Table 1:** summary of findings using the 5C model

5Cs: Construct	Theme	Quotes
Confidence	Vaccine efficacy concerns	“*But there are fears…a lot of information we are getting is that it does not protect you from acquiring COVID-19. So, why must I get vaccinated if it is not going to protect me?*” **(Male, Laboratory technologist, IDU)**
	Vaccine safety concerns	“*Its development was rushed. Much is not known about it in terms of its long-term effects. And there is not much information given about the vaccine, which makes people have a lot of conspiracy theories about it*” **(Female, Nurse, non-IDU- 020)**
	Mistrust in the healthcare system	“*When other people are allowed to import (COVID-19 vaccine) it… we won't know whether it is the right one or which one…corruption at bodies mandated to look at matters' health*” **(Male, Medical officer, non-IDU)**
Convenience/Constraints	Uncertainties about second vaccine dose availability	“*If I get the first vaccine and I miss the second one what will happen? You see they need that assurance, that if I get the first dose will I get the second one?*” (**Female, Laboratory technologist, IDU)**
	Inadequate vaccine knowledge	“*I have tried to look for information from those who are giving the vaccine and them also they don't know. So, it is a bit difficult like you are advising somebody, what am I supposed to do, like what else am I also supposed to do before going for the vaccine*” **(Female, Clinical officer, non-IDU)**
	Misinformation	“*I think the falsehood that is being spread in social media, people are having issues of infertility that have not been proven yet*” **(Female, Medical officer, non-IDU)**
Complacency	COVID-19 infection or contact history	“*Yes, I got COVID and the symptoms were not serious but now I see my colleagues getting vaccinated and some of them went up in the ward on oxygen. So, I am afraid, I don't want to get up in a ward; so, I'm banking on the immunity I acquired when I got COVID for now. yeah*.” **(Male, Social worker, non-IDU)**
Calculation	Seeking information before making decisions as to vaccination	“*Yeah, so initially they were fearful of the vaccination and then they wanted to see…if we would have any adverse effects. So, after we had spent a month or two after we had been immunized, they decided to go for the vaccine*” **(Male, Nurse, non-IDU)**
Collective response	Religious beliefs and conspiracy theories	“*Some of us are totally against it based on their religion…I mean maybe some were taught about it especially the Catholics. I think the catholic church is mostly against vaccines so some of them I know up to now have never taken the vaccine but others were okay about it*.” **(Male, Nurse, IDU)**

Low confidence in the COVID-19 vaccine including mistrust in the healthcare system and government, vaccine safety, and efficacy concerns were mentioned by health providers as reasons for vaccine hesitancy. They reported that they were hesitant to receive the vaccine as they perceived its development process was rushed and that the reports from the media highlighted the vaccine's potential and experienced side effects from people who got vaccinated. Additionally, constraints such as uncertainty about the second vaccine dose availability, misinformation, and inadequate vaccine knowledge were mentioned as reasons for poor vaccine uptake. Further, health providers were reluctant to receive the vaccine because of perceived herd immunity from a recent COVID-19 infection, religious and conspiracy theories, and seeking information before deciding to receive the vaccine. Health providers suggested the need to have well-planned strategies in place to ensure a reliable supply of the vaccine and that public sensitization of new vaccines should be prioritized before roll-out to improve uptake.

Our study findings underscore significant public health challenges regarding vaccine hesitancy among health providers, which can impact broader vaccination efforts. Safety concerns were mostly mentioned by health providers in this study as reasons for COVID-19 vaccine hesitancy. They attributed the vaccine's safety concerns to its perceived quick development compared to other vaccines and its potential side effects from media reports. Therefore, there is a need to ensure health providers' vaccine concerns and medicine adverse effects are considered before rollout programs. A review of evidence-based strategies to increase vaccination uptake highlighted that not all events occurring after vaccination can be attributed to the vaccine itself. The review emphasized the importance of ensuring access to medical advice when adverse events occur because this is crucial in building trust and addressing any concerns that may arise before it increases fear and escalates the overall negative experience [4].

Misinformation was also a key reason for vaccine hesitancy. Other studies have demonstrated that if misinformation remains unaddressed, it can affect individuals' perceptions and decision making leading to self-perpetuating negative news [5,6]. However, providing adequate and correct information to people with high levels of anxiety may not necessarily improve vaccine uptake therefore, alternative remedies need to be identified [7]. This reiterates the need to recognize that reasons for vaccine hesitancy are often complex and go beyond the scope of information alone. Therefore, the role of health providers, religious leaders, and public health officials in disseminating correct vaccine information is important but not adequate.

## Conclusion

Health providers are seen as trusted sources of information about vaccines. When they express hesitancy, it can erode public trust in vaccines in general, not just COVID-19 vaccines. This can lead to decreased uptake of routine vaccines among them, clients, and the general population. Therefore, there is a need to address individual, community, and religious concerns regarding any new vaccine before roll-out as this is likely to increase uptake among health providers, clients, and the general population.
